# Development and Application of EST-SSR Markers in *Cephalotaxus oliveri* From Transcriptome Sequences

**DOI:** 10.3389/fgene.2021.759557

**Published:** 2021-11-17

**Authors:** Hanjing Liu, Yuli Zhang, Zhen Wang, Yingjuan Su, Ting Wang

**Affiliations:** ^1^ School of Life Sciences, Sun Yat-sen University, Guangzhou, China; ^2^ Research Institute of Sun Yat-sen University in Shenzhen, Shenzhen, China; ^3^ College of Life Sciences, South China Agricultural University, Guangzhou, China

**Keywords:** *Cephalotaxus oliveri*, transcriptome, EST-SSRs, genetic diversity, population structure, transferability

## Abstract

*Cephalotaxus oliveri* is an endemic conifer of China, which has medicinal and ornamental value. However, the limited molecular markers and genetic information are insufficient for further genetic studies of this species. In this study, we characterized and developed the EST-SSRs from transcriptome sequences for the first time. The results showed that a total of 5089 SSRs were identified from 36446 unigenes with a density of one SSR per 11.1 kb. The most common type was trinucleotide repeats, excluding mononucleotide repeats, followed by dinucleotide repeats. AAG/CTT and AT/AT exhibited the highest frequency in the trinucleotide and dinucleotide repeats, respectively. Of the identified SSRs, 671, 1125, and 1958 SSRs were located in CDS, 3′UTR, and 5′UTR, respectively. Functional annotation showed that the SSR-containing unigenes were involved in growth and development with various biological functions. Among successfully designed primer pairs, 238 primer pairs were randomly selected for amplification and validation of EST-SSR markers and 47 primer pairs were identified as polymorphic. Finally, 28 high-polymorphic primers were used for genetic analysis and revealed a moderate level of genetic diversity. Seven natural *C. oliveri* sampling sites were divided into two genetic groups. Furthermore, the 28 EST-SSRs had 96.43, 71.43, and 78.57% of transferability rate in *Cephalotaxus fortune*, *Ametotaxus argotaenia,* and *Pseudotaxus chienii*, respectively. These markers developed in this study lay the foundation for further genetic and adaptive evolution studies in *C. oliveri* and related species.

## Introduction

Usually, successful conservation strategies require obtaining the genetic information, of which genetic diversity and population structure are essential parts. Molecular markers become useful tools to study genetic diversity and population structure of natural germplasm resources in non-model plants with no reference genomes (Parida et al., 2009). Compared to the other makers, SSRs possess the advantages of relative abundance, high polymorphism, codominant inheritance, and reproducibility, which have gained considerable importance in plant genetics and breeding (Taheri et al., 2018). In addition, SSRs have also been applied for discovering quantitative trait loci (QTL), linkage map construction between gene and marker, marker assisted selection for desired traits (MAS), and so forth (Kalia et al., 2011).

According to the source, SSRs are classed into genomic SSRs (gSSRs) and expressed sequence tag SSRs (EST-SSRs), the latter of which are located in the genic transcribed regions and are identified by NGS technology (Varshney et al., 2005; Wei et al., 2011). In general, EST-SSRs have been found to be more development-inexpensive, more evolutionarily conserved, and have higher transferability to related species than traditional anonymous gSSRs (Scott et al., 2000; Gadissa et al., 2018). Moreover, they are expected to be less polymorphic than gSSRs because of greater DNA sequence conservation in transcribed regions, but also less prone to null alleles (Postolache et al., 2014). The location of EST-SSRs determines their functional roles. EST-SSRs in CDSs affect the inactivated or activated genes or truncate proteins, in 3′UTR are involved in gene silencing or transcription slippage, and in 5′UTR impact gene transcription and/or translation (Lawson and Zhang, 2006; Gao et al., 2013).

*Cephalotaxus oliveri* (Cephalotaxaceae) is an endemic conifer species of China. It is a perennial shrub or small tree which is 4 m tall with white stomatal bands between the midvein and marginal bands of abaxial leaves. This plant can be cultivated as an ornamental in gardens, and its wood can be used for the manufacture of farm tools and furniture (Fu et al., 1999). Moreover, a variety of plant alkaloids (such as anticancer alkaloid harringtonine) can be extracted from leaves, branches, seeds, and roots, which have certain curative effects on leukemia and lymphoid sarcoma (Ni et al., 2016; Xiao et al., 2019). The species is distributed in broad-leaved or coniferous forests to the south of Qinling mountains–Huaihe River line and west of Wuyi Mountains at an altitude of 300–1800 m, and the distribution locations mainly include southeastern and northeastern Yunnan, Guizhou, southern and western Sichuan, northwestern Hubei, Hunan, eastern Jiangxi, and northern Guangdong (Fu et al., 1999). However, its natural population sizes decreased significantly in the recent years due to overexploitation, deforestation, and climate change. This species has been listed as vulnerable in the IUCN Red List. Thus, effective strategies are important to ensure the conservation and scientific utilization of *C. oliveri.* EST-SSRs have been widely developed and applied to numerous coniferous species. Zhang et al. (2015) developed nine EST-SSRs of *Larix gmelinii* and used them to evaluate the genetic parameters and transferability to three related species. Zeng et al. (2018) developed 11 EST-SSRs of *T. grandis*, which revealed a moderate level of genetic diversity and two different genetic groups within this species. Li et al. (2021) also investigated the genetic variation and population structure of 20 EST-SSRs. All these studies proved that EST-SSRs are effective molecular markers for studying the genetic diversity of conifers with high transferability. Although, hitherto, few SSRs (Pan et al., 2011; Miao et al., 2012) and ISSRs (Wang et al., 2016) have been developed and applied, there have been no reports on EST-SSR markers for *C. oliveri,* even for Cephalotaxaceae, using transcriptome data, greatly limiting research on genetic diversity, germplasm preservation, and molecular breeding of this species.

In this study, we used the leaf transcriptome data by Illumina sequencing from *C. oliveri*, and the objectives were to *1*) characterize frequency, distribution, and function of the SSR motifs from transcriptome unigenes; *2*) develop and characterize novel EST-SSRs and examine the level of polymorphism; *3*) analyze the cross-species transferability of the polymorphic EST-SSRs; and *4*) explore the genetic diversity and structure of *C. oliveri* by polymorphic EST-SSRs.

## Materials and Methods

### Plant Materials and DNA Extraction

In 2019, 134 *C. oliveri* individuals from seven natural sampling sites in China were collected for development and characterization of EST-SSRs; detail sample information is shown in Supplementary Figure S1 and Supplementary Table S1. Young leaves were sampled and placed into sealed bags containing dry silica gel and stored at 20°C for later use. The distance between individuals was more than 10 m. Total genomic DNA was extracted using the modified cetyltrimethylammonium bromide (CTAB) method (Su et al., 2005). DNA quality and concentration were determined by 1% agarose gel electrophoresis and Nanodrop 2000c (Thermo scientific, MA, United States), respectively. Then, all samples of DNA were diluted to a desired working concentration (50 ng/μl) and maintained at −20°C for PCR amplification.

Our sampling study complies with the laws of the People’s Republic of China. Voucher specimens were maintained at the Herbarium of Sun Yat-sen University (No: bzsjs-2019-1001∼bzsjs-2019-1007, bds-2018-1001, shs-2017-1001, sjs-2020-1001).

### EST-SSR Detection and Primer Design

The unigenes of leaf transcriptome data used for SSR development in this study came from the study conducted in our laboratory by He et al. (He et al., 2021) (Accession Number: SRR12058210). Potential SSRs were detected from 36446 unigenges using the the microsatellite tool (MISA, http://pgrc.ipk-gatersleben.de/misa/misa.html). The identification criteria for SSRs were set at a minimum number of 10, 6, 5, 5, 5, and 5 repeat units for mono, di, tri, tetra, penta, and hexanucleotide motifs, respectively. Primers were designed using Primer 3 software with major parameters as follows: primer length of 18–27 bp, PCR product size ranging from 100 to 280 bp, GC content of 40–60%, and annealing temperature ranging from 57 to 62°C.

### Functional Annotations and Classification of EST-SSRs

All unigenes containing SSRs were compared against six public databases by BLAST, including NCBI nonredundant protein sequences (NR), NCBI nonredundant nucleotide sequences (NR), Kyoto Encyclopedia of Genes and Genomes (KEGG), SWISS-PROT, Protein family (Pfam), and Clusters of eukaryotic Orthologous Groups (KOG). Blast2GO was used to perform Gene Ontology (GO) terms based on the Nr annotation (Conesa et al., 2005).

### EST-SSR Amplification and Validation

Totally, 238 pairs of primers were randomly selected (SSRs containing mononucleotides were not considered because of the high error rate of PCR product) and synthesized by TSINGKE Biological Technology Co., Ltd. (Beijing, China) for polymorphic EST-SSR development. PCR amplification was performed in a total reaction volume of 25 μl that included 1 μl of template DNA (50 ng/μl), 12.5 μl of 2×Taq Master Mix (Vazyme Biotech; Nanjing, China), 0.5 μl of each primer (10 μM), and 10.5 μl of ddH_2_O. Amplifications were carried out applying the following procedures: 5 min at 95°C for initial denaturation, followed by 30 cycles of 30 s at 95°C, 30 s at the annealing temperature, 30 s at 72°C, and 10 min at 72°C for final extension. The successfully amplified products were processed in 6% denaturing polyacrylamide gel electrophoresis for polymorphism screening by 14 individuals from seven sampling sites of *C. oliveri*. Polymorphic primers were further used for genotyping, and the PCR products with fluorescence labeling were separated on an ABI 3730xl DNA Analyzer (Applied Biosystems, CA, United States), using GeneScan LIZ500 (Applied Biosystems) as the internal lane size standard. The genotyping data were obtained through GeneMapper v5 (Applied Biosystems).

### Analysis of Data

We used the 28 EST-SSR markers to analyze genetic diversity and structure among 134 individuals from seven sampling sites. POPGENE v1.32 (Yeh et al., 1999) was used to calculate population genetic parameters, including the number of alleles (A), number of effective alleles (Ae), observed heterozygosity (Ho), expected heterozygosity (He), and Shannon’s information index (I). The polymorphism information contents (PICs) of each marker were calculated using PIC_CALC v0.6 (Botstein et al., 1980). MICRO-CHECKER 2.2.3 (Van Oosterhout et al., 2004) was used to estimate the null allele frequency for each marker. Linkage disequilibrium (LD) for locus pair and the departure from Hardy–Weinberg equilibrium (HWE) were detected by GENEPOP v4.7 ((Raymond and Rousset, 1995), Bonferroni corrections were performed to determine significance levels for all tests at *p-value* < 0.05 (Rice, 1989). Analysis of molecular variance (AMOVA) and principal coordinate analysis (PCoA) were performed in GenAlEx v6.5 (Peakall and Smouse, 2012).

Based on Bayesian clustering analysis, STRUCTURE v2.3.4 (Pritchard et al., 2000) with the default setting of the admixture model was used to analyze the genetic structure of natural populations within the species. Ten independent runs were performed with K from 1 to 10. Each run was estimated with Markov chain Monte Carlo steps of 100,000 iterations and burn-in period of 100,000 iterations. The optimal K value was evaluated in STRUCTURE HARVESTER (http://taylor0.biology.ucla.edu/structureHarvester/). CLUMPP (Jakobsson and Rosenberg, 2007) and Distruct (Rosenberg, 2004) were used to estimate the averaged admixture coefficients for each K value and visualize the clustering results, respectively.

### Transferability in Cross-Species

Three different species, namely, *C. fortunei*, *Ametotaxus argotaenia,* and *P. chienii* were used to analyze the transferability of the 28 EST-SSR markers. Young leaves of 17, 12, and 12 individuals were collected. Genomic DNA extraction, PCR amplification, and separation and size reading of target products for all these samples were performed as described above.

## Results

### Frequency and Distribution of EST-SSRs

**FIGURE 1:**
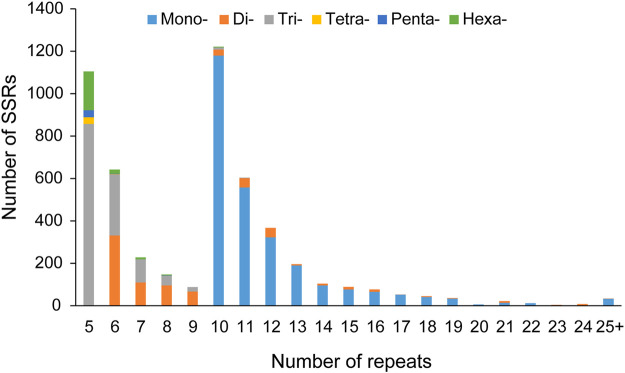
Number of repeats in each different unit length in *C. oliveri*.

Of the 36446 unigenes that we identified, 4352 sequences contained 5089 SSRs with 261 sequences in compound formation and 578 sequences containing more than 1 SSR. An overall density of 1 SSR/11.1 kb of the sequences was determined (Supplementary Table S2). The mononucleotides (2686, 52.78%) were the most abundant type of repeat motifs, followed by trinucleotides (1333, 26.19%), dinucleotides (781, 15.35%), hexanucleotides (219, 4.30%), pentanucleotides (36, 0.71%), and tetranucleotides (34, 0.67%), with the number of repeat units from 5 to 82. The largest number of repeat units was 10 (1222, 24.01%), followed by 5 (1105, 21.71%), 6 (642, 12.62%), 11 (604, 11.87%), and 12 (367, 7.21%). Most (99.33%) of the motifs had 5–24 repeats, while motifs with more than 24 repeats only accounted for 0.67% (Figure 1).

Among 109 different repeat motifs, A/T (2656, 52.19% of all motifs) was the most abundant motif in mononucleotide repeats, followed by C/G (30, 0.59%). In dinucleotide repeats, the most abundant type was AT/AT (536, 10.53%) followed by AG/CT (168, 3.30%). Among the trinucleotide repeats, the most frequent motif was AAG/CTT (331, 6.50%), followed by AGC/CTG (302, 5.93%) and AGG/CCT (236, 4.64%). AAAT/ATTT (8, 0.16%), AAGGC/CCTTG (10, 0.20%), and AAGAGG/CCTCTT (15, 0.29%) were identified as the top motifs in the hexanucleotide, pentanucleotide, and hexanucleotide repeats, respectively (Table 1).

**TABLE 1:** Frequencies of different repeat motifs in *C. oliveri* SSR loci.

Repeats	5	6	7	8	9	10	11	12	13	14	15	16	17+	Total	Rank	%
A/T						1165	553	320	188	95	75	66	194	2656	1	52.19
C/G						15	5	3	2	1	2		2	30	14	0.59
AC/GT		46	16	5		4	1	1	1			1		75	10	1.47
AG/CT		92	25	16	5	5	15	4		1			5	168	6	3.30
AT/AT		191	69	75	62	19	28	39	5	7	12	10	19	536	2	10.53
CG/CG		2												2	32	0.04
AAC/GTT	53	11	2	4			2							72	11	1.41
AAG/CTT	221	69	28	7	3	3								331	3	6.50
AAT/ATT	84	38	11	5		1								139	8	2.73
ACC/GGT	43	15	6	4	3	1								72	12	1.41
ACG/CGT	6			4	4									14	16	0.28
ACT/AGT	7													7	24	0.14
AGC/CTG	201	62	21	10	8									302	4	5.93
AGG/CCT	138	57	24	9	3	5								236	5	4.64
ATC/ATG	68	19	11											98	9	1.93
CCG/CGG	36	17	6	3										62	13	1.22
AAAC/GTTT	7													7	25	0.14
AAAT/ATTT	8													8	22	0.16
AAGGC/CCTTG	10													10	18	0.20
ACCCC/GGGGT	6													6	26	0.12
AACGGC/CCGTTG	1	3	2											6	27	0.12
AAGAGG/CCTCTT	15													15	15	0.29
AAGATG/ATCTTC	5													5	31	0.10
AAGCAG/CTGCTT	5	1												6	28	0.12
AAGGTG/ACCTTC	4			2										6	29	0.12
AATCAC/ATTGTG		4		1		1								6	30	0.12
ACCTCC/AGGTGG	6					3								9	20	0.18
AGAGGC/CCTCTG	8	2												10	19	0.20
AGATGG/ATCTCC	7	1												8	23	0.16
AGCAGG/CCTGCT	7	2												9	21	0.18
AGCCTC/AGGCTG	12													12	17	0.24
Others	147	10	7	2										166	7	3.26
Total	1105	642	228	147	88	1222	604	367	196	104	89	77	220	5089		100.00

**FIGURE 2:**
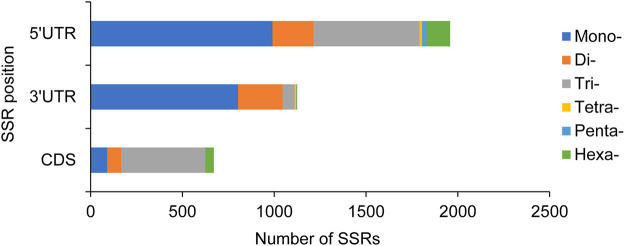
Distribution of six repeat motifs in different unigene positions.

In addition, the physical positions of these 5089 SSRs in the unigenes were also identified, and 671, 1125, and 1958 SSRs were located in CDS, 3′UTR, and 5′UTR, respectively, while the remaining 1335 SSRs had no sufficient information to determine their position. In CDS, trinucleotide repeats were the dominant type. Most of mononucleotide and dinucleotide repeats were located in 3′UTR and 5′UTR, whereas trinucleotide repeats were also abundant in 5′UTR (Figure 2).

### SSR Annotation and Classification

To explore the potential function of SSR-containing unigenes, all these unigenes were annotated by comparison against the seven functional databases: Nr, Nt, KEGG, Swiss-Prot, Pfam, KOG, and GO. As indicated in Table 2, a total of 3774 (86.72%) unigenes were annotated in at least one database and 584 (13.42%) were annotated in all databases. For each database, 3569 (82.01%) of unigenes were matched in Nr, 2076 (47.70%) in Nt, 1488 (34.19%) in KEGG, 2902 (66.68%) in SWISS-PROT, 2919 (67.07%) in Pfam, 2919 (67.07%) in GO, and 1188 (27.30%) in KEGG.

**TABLE 2:** Summary of functional annotation results of SSR-containing unigenes in *C. oliveri* transcriptome.

Annotated databases	Number of unigenes	Percentage (%)
Annotated in NR	3569	82.01
Annotated in NT	2076	47.70
Annotated in KO	1488	34.19
Annotated in SWISS-PROT	2902	66.68
Annotated in Pfam	2919	67.07
Annotated in GO	2919	67.07
Annotated in KEGG	1188	27.30
Annotated in all databases	584	13.42
Annotated in at least one database	3774	86.72
Total unigenes	4352	100.00

**FIGURE 3:**
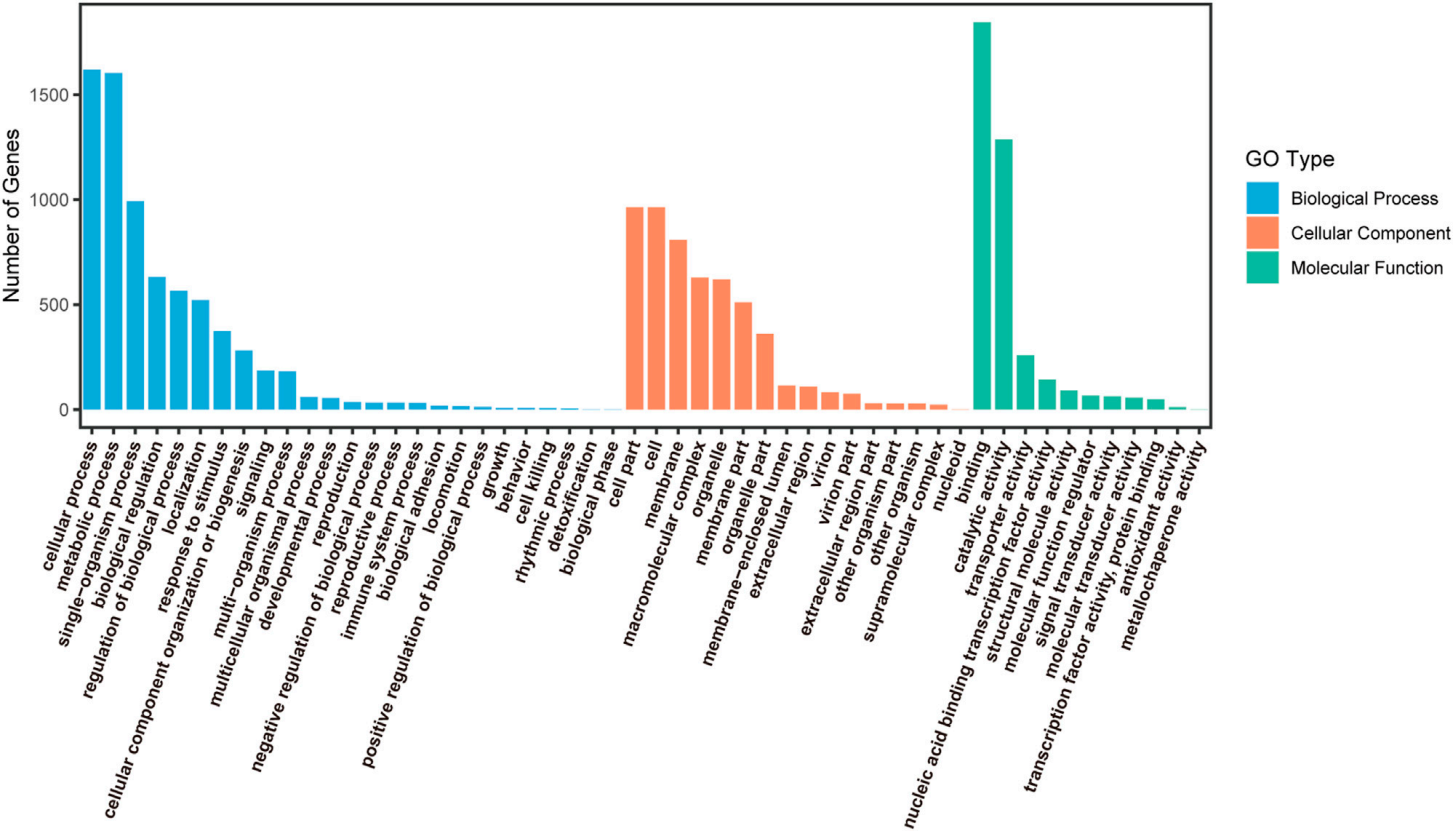
Level 2 GO classification of the annotated SSR-containing unigenes.

Classification of all SSR-containing unigenes was performed using their annotations with GO, KEGG, and KOG databases. These SSR-containing unigenes were classified to three major GO functional categories: biological process (7287, 44.14%), cellular component (5350, 32.40%), and molecular function (3874, 23.46%), which were further classified into 25, 16, and 11 different sub-categories, respectively (Figure 3). Of these, unigenes related to cellular process, metabolic process, and single-organism process accounted for the largest proportion in biological processes. In the cellular component, the most enriched sub-category was the cell part, followed by the cell and membrane. The molecular function category mainly represented the genes involved in binding, catalytic activity, and transporter activity.

**FIGURE 4:**
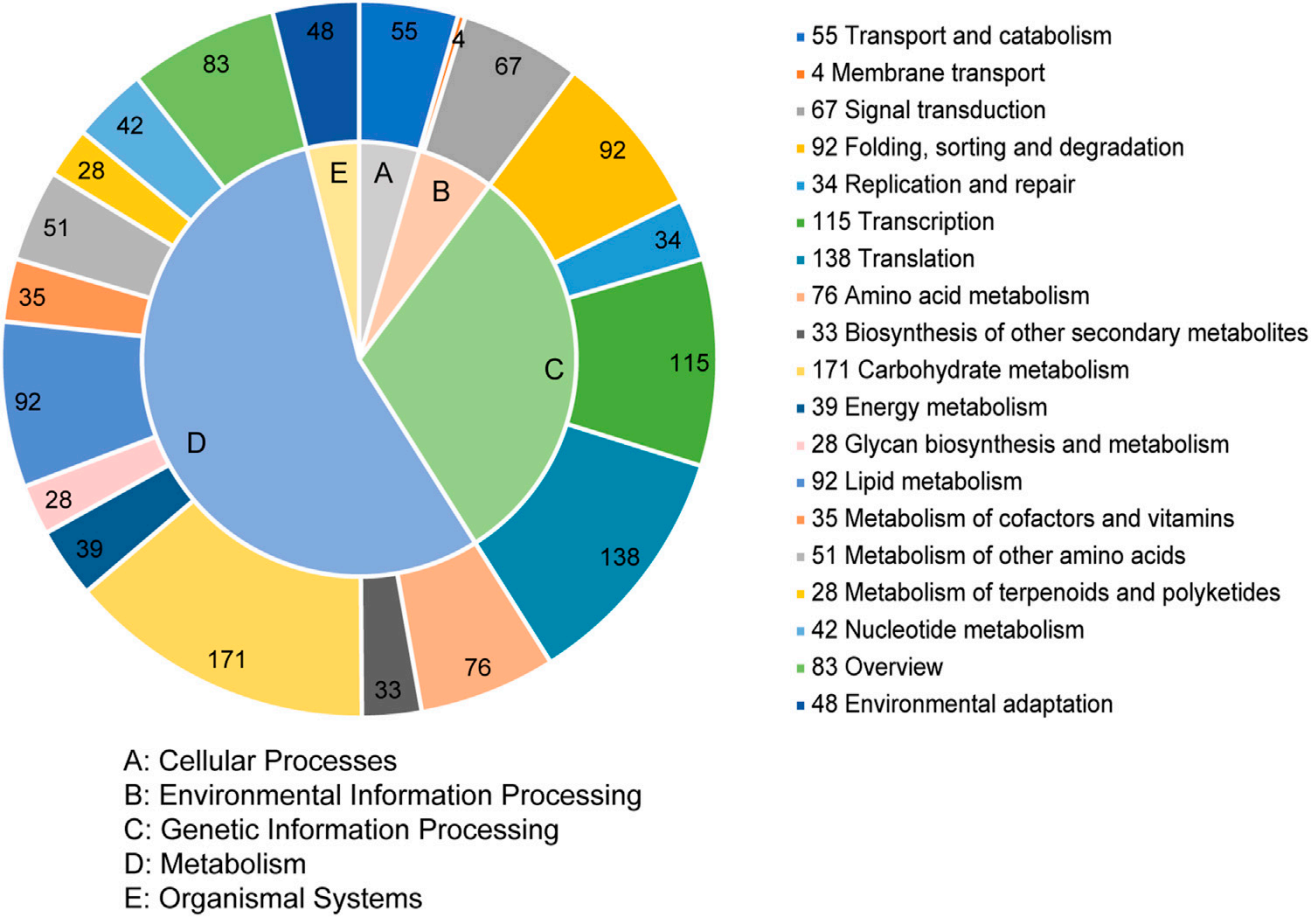
KEGG classification of the SSR-containing unigenes.

The unigenes were annotated in 115 KEGG metabolism pathways that were classified into five categories, including cellular processes (55, 4.47%), environmental information processing (71, 5.77%), genetic information processing (379, 30.79%), metabolism (678, 55.08%), and organismal systems (48, 3.90%) (Figure 4). In the second level of the pathway, carbohydrate metabolism represented the largest pathway, followed by translation and transcription. Among these 115 pathways, the top five were spliceosome, plant hormone signal transduction, starch and sucrose metabolism, mRNA surveillance pathway, and plant-pathogen interaction.

**FIGURE 5:**
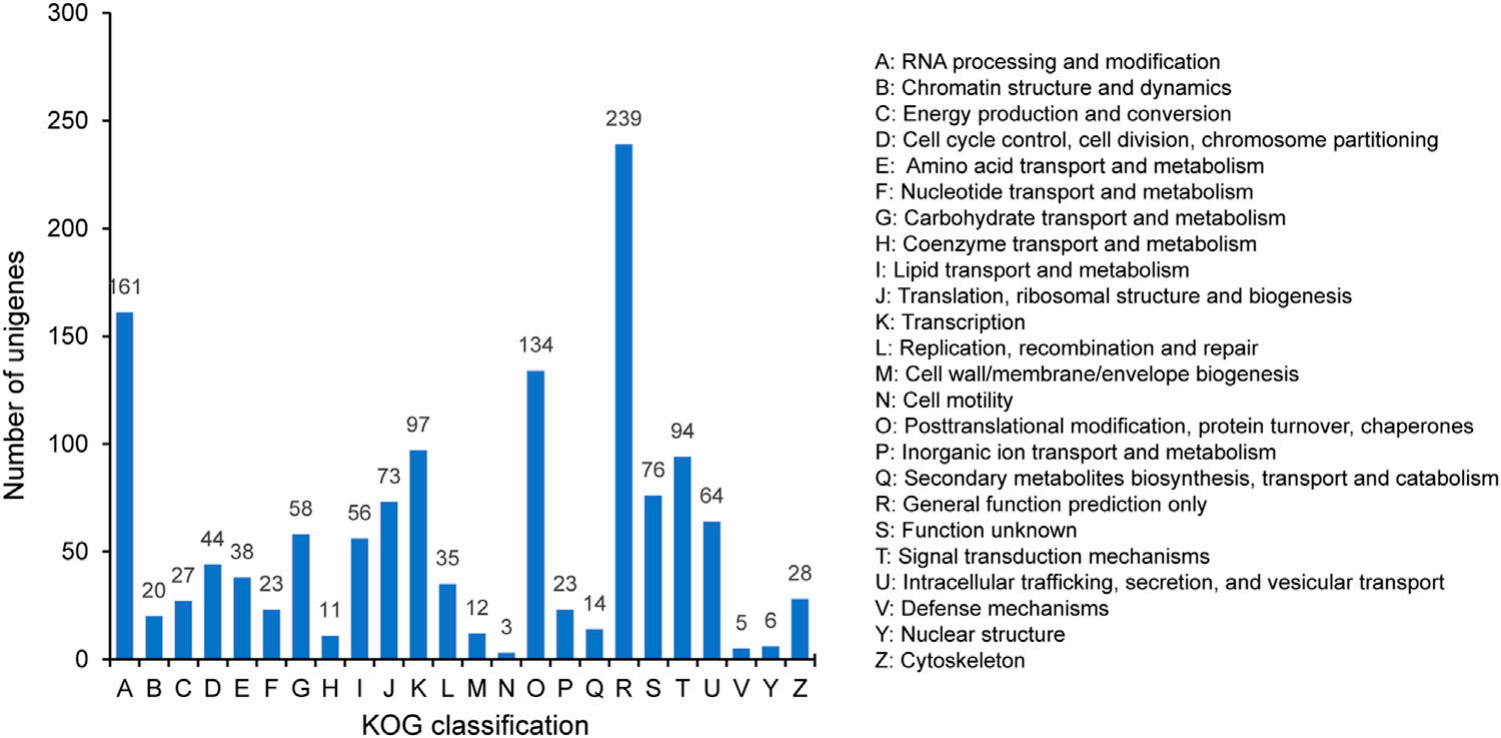
KOG classification of the SSR-containing unigenes.

The 1341 unigenes annotated by KOG were classified into 24 categories. The largest category was general function prediction only (239, 17.82%), followed by RNA processing and modification (161, 12.01%), posttranslational modification, protein turnover, chaperones (134, 9.99%), transcription (97, 7.23%), and signal transduction mechanisms (94, 7.01%) (Figure 5). The proportion in cell mobility (3, 0.22%) was the least, along with defense mechanisms (5, 0.37%) and nuclear structure (6, 0.45%).

### Development and Validation of EST-SSR Markers

Among the 5089 SSRs, 3900 pairs of primers were successfully designed. 238 pairs of primers were randomly selected for amplification and validation, and the results showed that 127 (53.36%) primers produced expected size bands. Of these 127 pairs of primers, 80 were monomorphic and 47 were identified as polymorphic. Finally, 28 high-polymorphic primers were selected and used for genetic analysis (Table 3).

**TABLE 3:** Characteristics of 28 newly developed polymorphic EST-SSR markers in *C. oliveri*.

Locus	Primer sequences (5′-3′)	Repeat motif	Allele size (bp)	T (°C)	Fluorescent dye	A	Ae	I	Ho	He	PIC	Null
Co258	F: AGG​AGC​CGG​AAC​AGG​AAA​TG	(TGGAAA)5	99–123	61	FAM	4	1.137	0.281	0.097	0.121	0.116	-0.1569(N)
	R: AAA​CAC​AAT​GTG​CTC​CAC​GC											
Co229	F: TGC​TTG​GCG​GGT​ATG​ATG​TT	(TGATTG)6	240–276	61	HEX	7	4.595	1.676	0.575	0.785	0.752*	0.3629(Y)
	R: AGG​TCA​GGA​TTC​GGG​AGG​AA											
Co161	F: CGA​CGC​CCA​TGA​GAC​TCA​AT	(TGGCAG)5	165–177	61	FAM	2	1.619	0.570	0.202	0.384	0.309	-0.2366(N)
	R: CAG​CCT​CTC​CTA​CAA​CTG​GC											
Co235	F: TGC​ATG​CAG​GCT​AAG​TTG​GA	(AGG)6	244–262	61	HEX	5	1.602	0.789	0.149	0.377	0.356	-0.0513(N)
	R: TGC​TTC​CAC​AGA​GTC​GTG​TC											
Co268	F: TCT​TCA​ACA​ACC​GCG​GAA​GA	(CGC)6	168–177	61	FAM	4	1.709	0.685	0.246	0.416	0.346*	-0.2254(N)
	R: CAA​GGA​AAT​CCA​GGA​GGC​GT											
Co264	F: AAG​CCC​AAG​CGG​AGT​TAG​AG	(AG)10	230–238	61	HEX	5	3.176	1.331	0.597	0.688	0.641**	0.0503(N)
	R: TGC​ATC​TGT​TCC​ACC​AGG​TC											
Co266	F: CTG​GGT​GGG​ATT​GCT​CCA​AT	(GGAAGA)5	209–227	61	FAM	3	1.978	0.840	0.463	0.496	0.433	-0.6041(N)
	R: TGG​ACG​CAA​GAA​GCC​TTC​AT											
Co111	F: AGC​CCA​GAA​GAT​TTG​CAT​GGA	(AAAC)5	245–269	61	HEX	4	3.072	1.171	0.612	0.677	0.606**	0.0115(N)
	R: TGA​GGT​CTG​CGT​TTG​AGG​AC											
Co146	F: GAT​CGG​CAA​TGC​TAA​TGG​CG	(TGCCAA)6	197–215	61	HEX	4	1.377	0.499	0.261	0.275	0.244*	-0.1633(N)
	R: TGT​TTC​TCT​CTG​CAC​CGG​TC											
Co267	F: GGA​CGA​CAG​CAA​TGG​CAT​TC	(CAG)8	204–225	61	HEX	5	1.087	0.224	0.067	0.080	0.079**	0(N)
	R: GGC​GAA​TGT​GTT​GCT​GGT​TT											
Co271	F: AAG​TTG​CAG​AAG​AGG​AGG​GG	(GAGGAA)5	149–161	61	FAM	3	1.231	0.351	0.090	0.189	0.171	0(N)
	R: CCT​CCT​CTG​CTT​CCT​CCT​CT											
Co274	F: TGG​CCT​GAT​ACG​ATT​GTG​CT	(TTAT)5	243–251	61	HEX	2	1.275	0.373	0.052	0.217	0.193**	0.4071(Y)
	R: TGT​GTG​AAA​AGG​AGC​CCG​AA											
Co75	F: GTG​GAA​CCT​CAG​TCT​GCG​AA	(AGG)7	174–183	61	FAM	3	1.136	0.266	0.097	0.120	0.115**	0(N)
	R: CAC​TCA​TCT​TCC​GCC​TCC​TC											
Co20	F: TAC​TGG​TCC​TCC​TAG​GCC​AC	(CCA)6	258–264	61	HEX	2	1.143	0.246	0.105	0.126	0.118	0(N)
	R: GCC​CAT​CTG​AGT​TCC​AGG​AG											
Co14	F: TGT​TGG​GGC​GGA​ATA​AGC​AT	(CCGTTG)6	255–279	61	FAM	5	2.930	1.273	0.455	0.661	0.610	0.1522(N)
	R: AGA​GCA​CTG​GTT​GAT​GGC​AA											
Co82	F: ACA​AGT​GTA​CGT​GTG​GCC​AA	(TCT)6	231–240	61	HEX	4	1.449	0.554	0.269	0.311	0.272**	0.2369(Y)
	R: TCT​AAC​GCA​GGA​CAG​ATC​GC											
Co43	F: TAA​CCT​AAG​AGG​GAG​GGG​GC	(GAC)8	246–267	61	FAM	6	1.157	0.361	0.075	0.136	0.133	0(N)
	R: GGG​CGT​CTG​GGA​TCC​ATT​AG											
Co22	F: AGC​CCA​GAA​GAT​TTG​CAT​GGA	(AAAC)5	245–269	61	HEX	4	3.169	1.240	0.530	0.687	0.625**	-0.1077(N)
	R: TGA​GGT​CTG​CGT​TTG​AGG​AC											
Co228	F: TGG​TGT​GGT​GTG​GTG​TAC​TG	(GATG)5	244–248	61	FAM	2	1.118	0.216	0.052	0.106	0.100	0(N)
	R: CGA​CAC​CAC​AAC​GCC​TTT​TT											
Co257	F: GAT​CAA​TGC​TCC​CCT​GCT​GT	(CAC)8	191–206	61	HEX	5	1.556	0.752	0.328	0.359	0.336**	-0.0118(N)
	R: TCT​CCA​CTA​CGG​CAC​TCT​CT											
Co66	F: TTT​CCC​CAC​CTC​TTC​CCA​GA	(AGC)6	202–211	61	FAM	4	1.259	0.442	0.179	0.206	0.196**	-0.0381(N)
	R: CAC​TGC​TCT​CAC​TGC​CTT​CA											
Co224	F: GCC​TTC​AAC​CCG​TCA​ACA​AC	(AGGAGC)5	217–235	61	HEX	4	1.908	0.849	0.097	0.478	0.430**	0.1877(N)
	R: ACC​ATA​GTC​CGG​GTG​AGG​AA											
Co77	F: GCC​ACT​GTT​ATT​CTG​CAG​CG	(GCC)7	230–248	61	FAM	6	2.529	1.122	0.343	0.607	0.530**	0.0757(N)
	R: AGC​GAT​GGT​AAC​GAG​CCT​TC											
Co236	F: CCA​GAG​ACC​CCA​GCA​AGA​AG	(AGA)8	244–259	61	HEX	4	2.084	0.797	0.836	0.522	0.407**	-0.1859(N)
	R: CGA​AGG​AGG​TTT​TGG​AGG​CT											
Co222	F: GCA​GCG​CCA​TTA​TCA​AGT​GG	(TGGTAC)5	199–229	61	FAM	5	1.961	0.979	0.224	0.492	0.458**	-0.0513(N)
	R: GGG​TAT​CTG​CCT​CGT​CAC​AC											
Co244	F: GCT​GAA​ATG​GGG​GAC​TCC​AA	(GAA)6	272–281	61	HEX	4	1.180	0.333	0.000	0.153	0.145*	0.3416(Y)
	R: GAT​CTT​TGC​GCC​CTG​TTT​CC											
Co234	F: TGC​AAC​AGC​AGC​CAC​ATC​TA	(CAGCAA)5	143–185	61	FAM	6	1.591	0.805	0.336	0.373	0.353	-0.3675(N)
	R: GGC​ATT​CCT​TGT​GGC​TGT​TG											
Co261	F: ATC​GCT​TCA​TGG​CAT​TGT​GC	(AATGGG)5	249–279	61	HEX	6	2.883	1.253	0.463	0.656	0.598**	-0.0679(N)
	R: TCA​GGA​TAG​GCT​TCC​TCC​GT											
Mean						4.214	1.890	0.724	0.279	0.382	0.345	

A, number of alleles; Ae, number of effective alleles; Ho, observed heterozygosity; He, expected heterozygosity; I, Shannon’s information index; PIC, polymorphic information content (*p < 0.05, significantly departures from Hardy–Weinberg equilibrium; **p < 0.01, extremely significantly departures from Hardy–Weinberg equilibrium); Null, the frequency of the null allele (Y, SSR, locus may have a null allele; N, SSR, locus may not have null allele).

In total, 118 alleles were detected in all samples by the 28 EST-SSR markers. A per locus ranged from 2 to 7 with an average of 4.214. Ae ranged from 1.087 to 4.595, with an average value of 1.889. I varied from 0.215 to 1.676, with an average value of 0.724. Ho and He were calculated as 0.279 and 0.382, ranging from 0.052 to 0.836 and 0.080 to 0.785, respectively. The PIC ranged from 0.079 to 0.752, with an average of 0.345. In addition, four loci (Co229, Co274, Co82, and Co244) were found to have null alleles. Significant departures from HWE were detected for 18 of 28 EST-SSR loci (Table 3). Among 378 pairs of loci, 44 pairs showed LD and only two (Co14&Co257 and Co229&Co222) remained significant after Bonferroni correction (*p-value* < 0.05), indicating that the EST-SSR loci used in this study were independent of each other.

### Genetic Diversity and Structure

For each sampling site, the mean A, Ae, I, Ho, and He ranged from 2.071 to 2.679, 1.451 to 1.720, 0.396 to 0.587, 0.208 to 0.311, and 0.243 to 0.352, respectively. Overall, SX had the greatest genetic diversity, whereas the lowest genetic diversity was found in PB (Supplementary Table S3). AMOVA showed that 71% of the variation was found within populations and 29% among populations (Supplementary Table S4).

**FIGURE 6:**
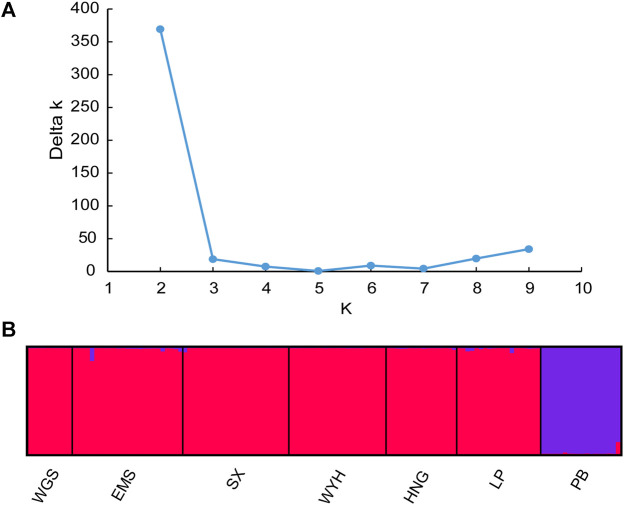
Structure analysis of *C. oliveri*. **(A)** Estimation of population using Delta k value with cluster K ranging from 1 to 10. **(B)** Estimation of the population structure based on STRUCTURE analysis.

**FIGURE 7:**
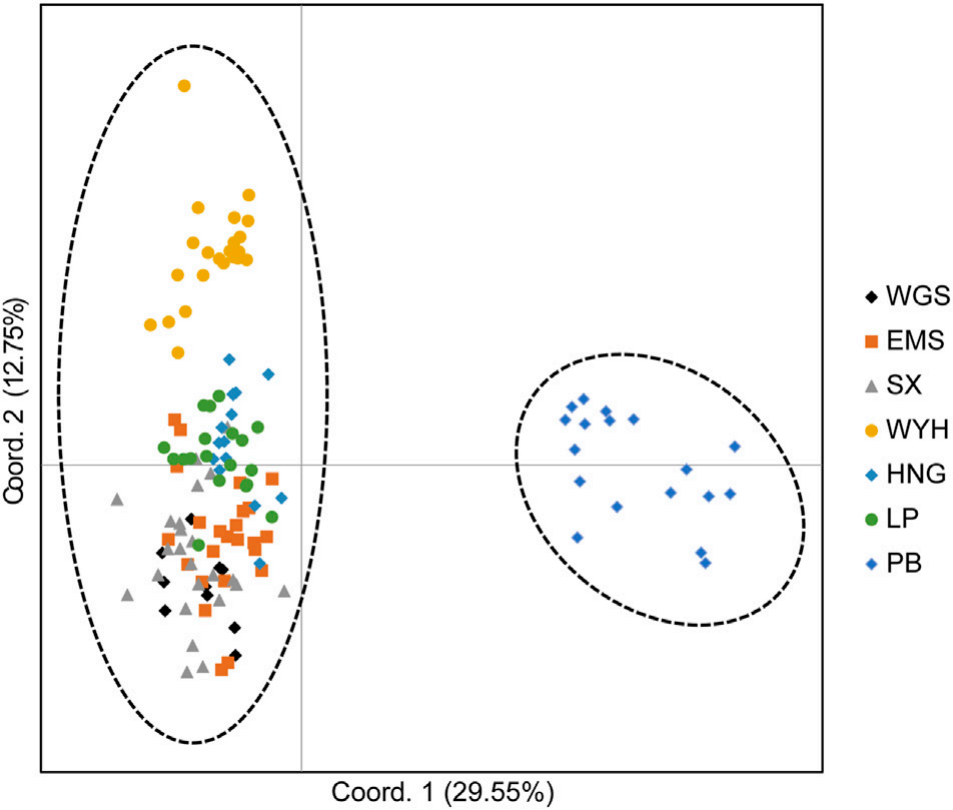
Principal coordinate analysis (PCoA) based on genetic distance.

The population structure of *C. oliveri* was analyzed in STRUCTURE, and the optimal K value was observed at K = 2 with maximum Delta K value (Figure 6A). The seven sampling sites were divided into two genetic groups (Figure 6B and Supplementary Figure S1). Group Ⅰ included the sites, namely, SX, HNG, WYH, LP, EMS, and WGS, while Group Ⅱ only included site PB. Consistent with the STRUCTURE analysis, the result of PCoA also revealed two groups based on genetic distance (Figure 7). The first and second axes explained 29.55 and 12.75% of the total variation, respectively. HWE was further tested for these two genetic groups, and the result showed that 13 loci deviated from HWE in Group Ⅰ, while only one locus was deviated from HWE in Group Ⅱ (Supplementary Table S5).

### Cross-Species Transferability

The 28 pairs of primers were also evaluated for transferability in the three species (*C. fortune*, *A. argotaenia* and *P. chienii*). The results showed that 15 primer pairs were successfully amplified in all species (all the templates). Twenty-seven pairs were applicable for (had transferability in) *C. fortunei*, with the highest rate of 96.43%. 20 (71.43%) and 22 (78.57%) pairs had transferability in *A. argotaenia* and *P. chienii*, respectively (Supplementary Table S6).

## Discussion

Currently, transcriptome sequencing through the Illumina platform is the most widely utilized NGS technology for EST-SSR marker development in non-model plants, particularly in conifers with large genomes (Zalapa et al., 2012; Postolache et al., 2014; Vieira et al., 2016). In this study, a total of 36446 unigenes were used to detect SSRs, and finally 4352 unigenes containing 5089 SSRs were identified. The distribution frequency was 11.94%, which was similar to that of *C. hainanensis* (11.39%) (Qiao et al., 2014) and *P. chienii* (11.15%) (Xu et al., 2020), higher than that of *P. bungeana* (9.21%) (Duan et al., 2017), *Torreya grandis* (2.75%) (Zeng et al., 2018), *A. argotaenia* (7.68%) (Ruan et al., 2019), and *P. koraiensis* (6.84%) (Li et al., 2021), lower than that of *Lycium barbarum* (27.93%) (Chen et al., 2017), *Dalbergia odorifera* (23.31%) (Liu et al., 2019), and peony (20.38%) (He et al., 2020). The average density of SSRs was 1/11.1 kb, lower than that of *C. hainanensis* (1/8.08 kb) (Qiao et al., 2014), *Glyptostrobus pensilis* (1/7.59 kb) (Li et al., 2019), and *P. chienii* (1/9.18 kb) (Xu et al., 2020), while higher than that in *P. dabeshanensis* (1/23.08 kb) (Xiang et al., 2015), *P. koraiensis* (1/17.38 kb) (Du et al., 2017), and *L. principis-rupprechtii* (1/26.8 kb) (Dong et al., 2018). The differences in frequency and density might be caused by several factors, including dataset size, SSR mining tools, search criteria, and genome structure (Varshney et al., 2005).

A microsatellite locus typically varies in length between 5 and 40 repeats, but longer strings of repeats are possible (Selkoe and Toonen, 2006). In this study, the repeat units of 10, 5, 6, 11, and 12 accounted for 77.42% of the total SSR loci, with the size mainly ranging from 10 to 18 bp. The predominant type was trinucleotide repeats (26.19%), followed by dinucleotide repeats (15.35%) and hexanucleotide repeats (4.30%). This result was similar to that of pervious reports that trinucleotide repeats were the abundant type for other conifers, including *C. hainanensis* (Qiao et al., 2014), *A. argotaenia* (Ruan et al., 2019), *P. koraiensis* (Li et al., 2020), and *P. chienii* (Xu et al., 2020). Among the trinucleotide repeats, the most abundant motif was AAG/CTT, which was identical to previous findings in *Cryptomeria japonica* (Ueno et al., 2012), *P. halepensis* (Pinosio et al., 2014), *L. gmelinii* (Zhang et al., 2015), *T. grandis* (Zeng et al., 2018), *A. argotaenia* (Ruan et al., 2019), and *P. chienii* (Xu et al., 2020). In addition, this motif was the second abundant motif in *C. hainanensis* (Qiao et al., 2014) and *P. dabeshanensis* (Xiang et al., 2015). These results showed that AAG/CTT was conserved in conifers. Of the dinucleotide repeats, AT/AT and AG/CT exhibited high frequency. However, like most conifers, including *T. contorta* (Majeed et al., 2019) and *P. koraiensis* (Li et al., 2020), the CG/CG motif was found to be rare in this study. It might be due to methylation of cytosine in CpG sequences, which might potentially inhibit transcription (Gonzalez-Ibeas et al., 2007; Xing et al., 2017).

Some studies have shown that SSRs are much more abundant in the UTRs than in the CDSs of many plants (Li et al., 2004). In the present study, 17.85% of SSRs were found to be located in CDSs in contrast to 82.13% in UTRs. Moreover, trinucleotide repeats were mostly accumulated in the CDSs and other types had a small proportion. This result was consistent with those of the studies in *T. contorta* (Majeed et al., 2019), *Elaeagnus mollis* (Liu et al., 2020), and *P. chienii* (Xu et al., 2020). This might explain that non-trinucleotides negatively selected frameshift mutations in coding regions, contributing to changes in SSR length, and therefore to expression. In contrast, trinucleotides did not generate frameshifts and did not affect gene expression through single-motif length mutations (Metzgar et al., 2000; Taheri et al., 2019).

All SSR-containing unigenes in leaves of *C. oliveri* were annotated through seven functional databases. 86.72% of these unigenes were annotated in at least one database, which was similar to the annotation result of all unigenes in leaf (He et al., 2021). In the GO classification, most of SSR-containing unigenes were categorized in the cellular process, metabolic process, single-organism process, cell part, cell, binding, and catalytic activity, which are involved in the basic metabolism in the plant cells. Furthermore, KEGG and KOG classification suggested that SSR-containing unigenes were involved in growth and development of *C. oliveri* with various biological functions.

In this study, among the 238 pairs of randomly designed primers, 127 (53.36%) were of expected size bands. Forty-seven (19.75%) were identified as polymorphic among 14 individuals from seven *C. oliveri* sampling sites. This polymorphism rate was higher than the results reported for *T. grandis* (10.38%) (Zeng et al., 2018), *P. koraiensis* (6.67%) (Li et al., 2020), *G. pensilis* (13.53%) (Li et al., 2019), and *P. chienii* (10.67%) (Xu et al., 2020), but lower than the results of *P. dabeshanensis* (Xiang et al., 2015) (23.17%) and *L. principis-rupprechtii* (21.67%) (Dong et al., 2018). In conifers with huge genome structure, most EST-SSRs might locate within repetitive DNA, which caused EST-SSRs difficult to be amplified and the polymorphism rate to be low (Wagner et al., 2012).

The transferability rate of the markers corresponds to genomic similarity and can reflect the genetic relationships and extent of sequence conservation between species (Zhang et al., 2014). EST-SSR markers are expected to have a high transferability rate due to conservation of transcribed regions among related species (Kalia et al., 2011). In this study, the transferability of 28 EST-SSRs from *C. oliveri* to *C. fortune* was 96.43%, confirming that *C. oliveri* had a closer relationship with *C. fortune* than the other two species. This transferability rate was consistent with that of *Taxodium 'zhongshansa'* (100%) (Cheng et al., 2015), while higher than that of *Abies alba* (75–81%) (Postolache et al., 2014) and *P. chienii* (70%) (Xu et al., 2020). Significantly, the transferability rate was more than 70% both for *A. argotaenia* and *P. chienii* belonging to Taxaceae, indicating that there may be a high genomic similarity between *C. oliveri* and Taxaceae species. These results indicated that the markers developed in this study would provide a powerful molecular tool for evolutionary adaptation and genetic relationship analysis in *C. fortune*, *A. argotaenia,* and *P. chienii*.

Genetic diversity is an important indicator in the conservation and management of plant genetic resources (Chen et al., 2018).In this study, genetic parameters were detected in 134 individuals by the 28 EST-SSRs. The mean PIC value was 0.345, indicating a mean level of genetic information, and similar results were observed in *C. japonica* (0.325) (Ueno et al., 2012), *A. argotaenia* (0.455) (Ruan et al., 2019), and *P. koraiensis* (0.404) (Li et al., 2021). Mean A (4.214) was higher than that reported for *T. grandis* (2.636) (Zeng et al., 2018) and *L. principis-rupprechtii* (3.850) (Dong et al., 2018), but lower than that for *P. koraiensis* (6.45) (Li et al., 2020) and *P. chienii* (6.4) (Xu et al., 2020). The average of Ho and He were 0.279 and 0.382, respectively, which was lower than that for *P. dabeshanensis* (0.445 and 0.486) (Xiang et al., 2015) and *L. principis-rupprechtii* (0.487 and 0.490) (Dong et al., 2018). These results revealed that the 134 individuals had a moderate level of genetic diversity, which might be caused by small population size and fragmentation; meanwhile, habitat heterogeneity might restrict gene flow between populations and had an impact on genetic diversity (Wang et al., 2014). However, this study on genetic diversity of *C. oliveri* showed a lower level than that in the previous study employing gSSRs (Ho = 0.570, He = 0.568) (Miao et al., 2012). These differences might be due to the different types and numbers of genetic markers used in the studies or to the different populations and sample sizes (Tong et al., 2020). Furthermore, the genetic diversity of the population level was lower than that at the species level, which might be due to limited numbers of SSR loci and small population size.

The distribution geographic range, habitat types, and species characteristics may have a significant influence on the population structure, as well as genetic diversity (Jia et al., 2016; Li et al., 2019). A previous report found that 22 populations of *C. oliveri* were clustered into two groups by constructing a dendrogram, and the YNdws population (Yunnan Province) was clustered into the separated group (Wang et al., 2016). In this study, STRUCTURE and PCoA analyses also revealed a distinct genetic population (site PB) among the seven sampling sites of *C. oliveri*. The main reason was that site PB was in the southern edge of the distribution area, as the most geographically distant population, which limited the gene flow between site PB and other sites. However, detailed reasons for the population structure need to be further investigated with an increased number of population and materials.

According to the results of genetic diversity and population structure of *C. oliveri*, we gained the following management implications and recommendations: for the sites (SX, LP) with higher genetic diversity, *in situ* conservation strategies should be designed to protect the habitats from human disturbance and prevent the loss of genetic diversity. *Ex situ* conservation programs should be carried out for the sites (EMS, PB) with lower genetic diversity in order to increase the species numbers of these sampling sites and improve their adaptability to the environment. Furthermore, special attention should be given to site PB because of its isolation from the other sampling sites; it is necessary to save the seeds of this site for breeding.

## Conclusion

In this study, 5089 EST-SSRs were identified from transcriptome data of *C. oliveri*, and the distribution, frequency, location, and function of motifs were characterized and evaluated., Twenty-eight EST-SSR markers were developed for *C. oliveri* with abundant polymorphisms and showed a moderate level of genetic diversity. Genetic structure and PCoA analysis revealed two different genetic groups of natural *C. oliveri*. In addition, more than 70% of these EST-SSRs could be transferred to other species. These EST-SSRs would enable further genetic investigation in *C. oliveri* and related species and could be used to ensure effective conservation and breeding applications of *C. oliveri* in the future.

## Data Availability

Publicly available datasets were analyzed in this study. These data can be found here: Twenty-eight EST-SSR sequences generated for this study were deposited at GenBank with Accession numbers MZ773613-MZ773640.
