# Supplementary material for: Development and Application of EST-SSR Markers in Cephalotaxus oliveri From Transcriptome Sequences
Source: Front Genet. 2021 Nov 17;12:759557. doi: 10.3389/fgene.2021.759557 (PMC8635753; doi:10.3389/fgene.2021.759557)
Supplement: Supplementary file 1 [file Table1.DOCX]

Supplementary Table 1 The sampling information of *C. oliveri* sites.

| Location | Site | Sample size | Latitude (N) | Longtitude (E) | Altitude  (m a.s.l) | Sampling date |
| --- | --- | --- | --- | --- | --- | --- |
| Sixi, Hubei | SX | 24 | 30°43′36.37″ | 110°54′43.53″ | 340 | 2019.6 |
| Hanigong, Hunan | HNG | 16 | 28°51′49.97″ | 109°55′06.50″ | 347 | 2019.6 |
| Wuyanghe, Guizhou | WYH | 22 | 27°3′24.64″ | 108°18′38.04″ | 508 | 2019.6 |
| Liangping, Chongqing | LP | 19 | 30°41′50.10″ | 107°34′17.58″ | 549 | 2019.6 |
| Emeishan,Sichuan | EMS | 25 | 29°33′12.39″ | 103°23′45.46″ | 976 | 2019.6 |
| Wugongshan, Jiangxi | WGS | 10 | 27°32′11.90″ | 114°10′31.10″ | 625 | 2019.7 |
| Pingbian, Yunnan | PB | 18 | 23°1′36.85″ | 103°42′22.16″ | 670 | 2019.7 |
