# Supplementary figures and images for: Development and Application of EST-SSR Markers in Cephalotaxus oliveri From Transcriptome Sequences
Source: Front Genet. 2021 Nov 17;12:759557. doi: 10.3389/fgene.2021.759557 (PMC8635753; doi:10.3389/fgene.2021.759557)

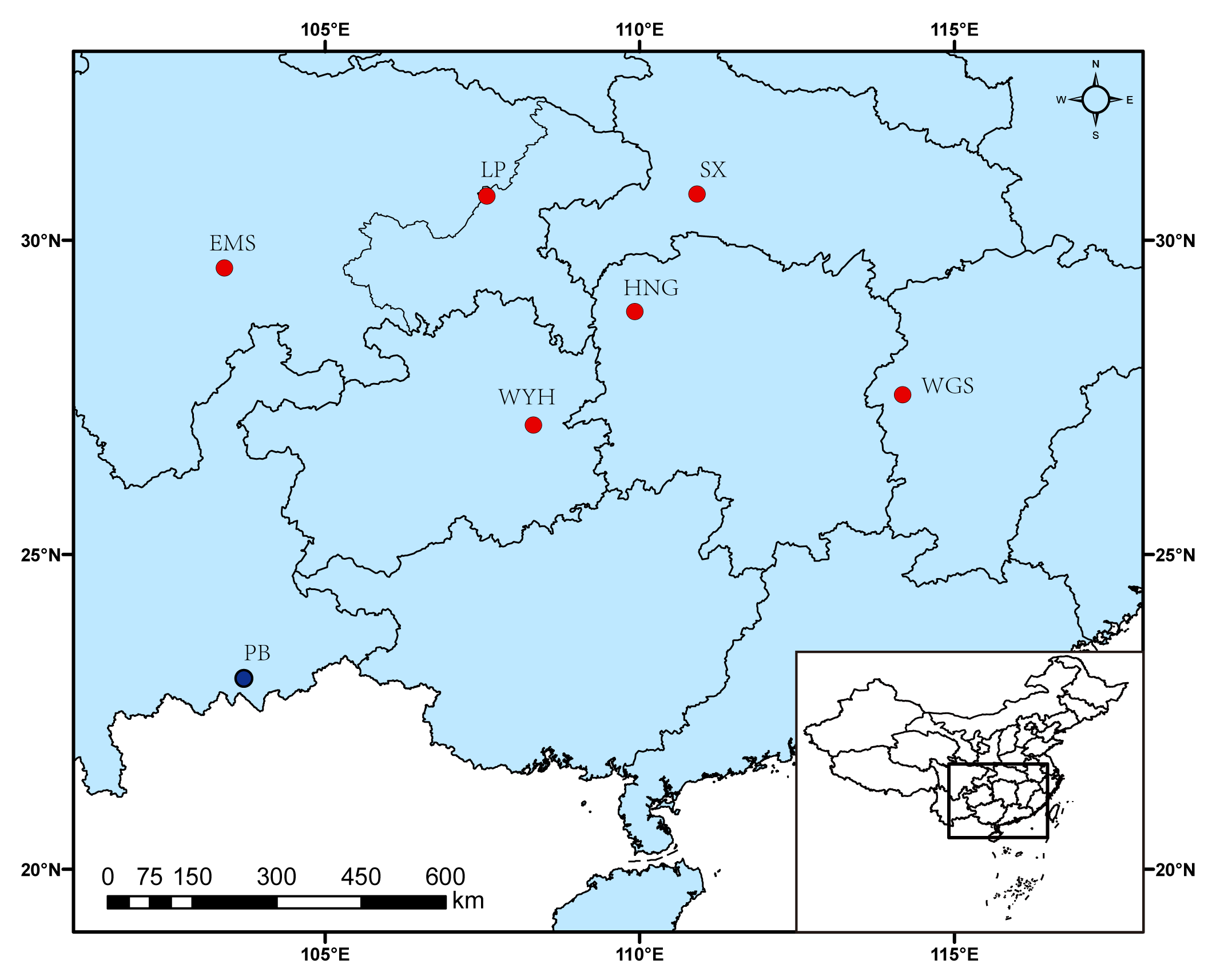

Supplement: Supplementary file 2 [file Image1.TIF]
