# Supplementary material for: Development and Application of EST-SSR Markers in Cephalotaxus oliveri From Transcriptome Sequences
Source: Front Genet. 2021 Nov 17;12:759557. doi: 10.3389/fgene.2021.759557 (PMC8635753; doi:10.3389/fgene.2021.759557)
Supplement: Supplementary file 3 [file Table2.DOCX]

Supplementary Table 2 Characteristics of unigenes and SSR in *C. oliveri* transcriptome.

| Sequence types | Total |
| --- | --- |
| Total number of sequences examined | 36446 |
| Total size of examined sequences (bp) | 56491341 |
| Total number of identified SSRs | 5089 |
| Number of SSR containing sequences | 4352 |
| Number of sequences containing more than 1 SSR | 578 |
| Number of SSRs present in compound formation | 261 |
| Frequency of SSR | 1/11.1 kb |
