# Supplementary material for: Development and Application of EST-SSR Markers in Cephalotaxus oliveri From Transcriptome Sequences
Source: Front Genet. 2021 Nov 17;12:759557. doi: 10.3389/fgene.2021.759557 (PMC8635753; doi:10.3389/fgene.2021.759557)
Supplement: Supplementary file 4 [file Table5.DOCX]

Supplementary Table 5 The result of Hardy-Weinberg test for two genetic groups

| locus | Group Ⅰ | Group Ⅱ |
| --- | --- | --- |
| Co258 | ns | - |
| Co229 | ns | ns |
| Co161 | ** | - |
| Co235 | * | ns |
| Co268 | ns | ** |
| Co264 | ** | ns |
| Co266 | * | ns |
| Co111 | ns | ns |
| Co146 | ns | ns |
| Co267 | ns | - |
| Co271 | ** | - |
| Co274 | ns | ns |
| Co75 | ns | - |
| Co20 | ns | - |
| Co14 | * | - |
| Co82 | ns | - |
| Co43 | ** | ns |
| Co22 | ** | - |
| Co228 | - | ns |
| Co257 | ns | ns |
| Co66 | ns | - |
| Co224 | ** | - |
| Co77 | ** | ns |
| Co236 | ** | ns |
| Co222 | ** | ns |
| Co244 | ** | ns |
| Co234 | ns | - |
| Co261 | ns | ns |

ns = not significant, * *P* < 0.05, ** *P* < 0.01
