# Supplementary material for: Development and Application of EST-SSR Markers in Cephalotaxus oliveri From Transcriptome Sequences
Source: Front Genet. 2021 Nov 17;12:759557. doi: 10.3389/fgene.2021.759557 (PMC8635753; doi:10.3389/fgene.2021.759557)
Supplement: Supplementary file 5 [file Table3.DOCX]

Supplementary Table 3 Genetic diversity parameters at the site level based on 28 EST-SSRs.

| Site | A | Ae | I | Ho | He |
| --- | --- | --- | --- | --- | --- |
| SX | 2.679 | 1.720 | 0.587 | 0.311 | 0.352 |
| HNG | 2.250 | 1.642 | 0.474 | 0.299 | 0.289 |
| WYH | 2.429 | 1.513 | 0.435 | 0.278 | 0.252 |
| LP | 2.750 | 1.680 | 0.549 | 0.323 | 0.310 |
| EMS | 2.143 | 1.563 | 0.425 | 0.230 | 0.256 |
| WGS | 2.071 | 1.511 | 0.441 | 0.332 | 0.287 |
| PB | 2.071 | 1.451 | 0.396 | 0.208 | 0.243 |
