# Supplementary material for: Development and Application of EST-SSR Markers in Cephalotaxus oliveri From Transcriptome Sequences
Source: Front Genet. 2021 Nov 17;12:759557. doi: 10.3389/fgene.2021.759557 (PMC8635753; doi:10.3389/fgene.2021.759557)
Supplement: Supplementary file 6 [file Table4.DOCX]

Supplementary Table 4 Analysis of molecular variance (AMOVA) in seven sites of *C. oliveri*.

| Source | d.f. | Sum of squares | Variance components | Percentage of variation |
| --- | --- | --- | --- | --- |
| Among sites | 6 | 388.307 | 1.603 | 29% |
| Within sites | 261 | 1039.805 | 3.984 | 71% |
| Total | 267 | 1428.112 | 5.587 | 100% |
