# Supplementary material for: Development and Application of EST-SSR Markers in Cephalotaxus oliveri From Transcriptome Sequences
Source: Front Genet. 2021 Nov 17;12:759557. doi: 10.3389/fgene.2021.759557 (PMC8635753; doi:10.3389/fgene.2021.759557)
Supplement: Supplementary file 7 [file Table6.DOCX]

Supplementary Table 6 Cross-species amplification of 28 EST-SSRs developed for *C. oliveri* in three related species

|  | *C. fortunei* (N=17) | | *A. argotaenia* (N=12) | | *P. chienii* (N=12) | |
| --- | --- | --- | --- | --- | --- | --- |
| Locus | Na | Allele size range (bp) | Na | Allele size range (bp) | Na | Allele size range (bp) |
| Co258 | 2 | 111, 117 | 2 | 117， 123 | 1 | 117 |
| Co229 | 1 | 239 | - | - | - | - |
| Co161 | 1 | 171 | 2 | 165， 177 | 3 | 165， 177 |
| Co235 | 3 | 237-246 | 1 | 241 | 2 | 244， 250 |
| Co268 | 3 | 171-177 | 2 | 174， 177 | 2 | 174， 177 |
| Co264 | 2 | 230 | - | - | 5 | 232-240 |
| Co266 | 2 | 209, 221 | 1 | 215 | 2 | 215， 211 |
| Co111 | 3 | 257-265 | 4 | 245-269 | 3 | 261-269 |
| Co146 | 2 | 185, 215 | 2 | 203， 209 | 2 | 205， 211 |
| Co267 | - | - | 1 | 213 | 2 | 212, 215 |
| Co271 | 2 | 149, 161 | - | - | - | - |
| Co274 | 2 | 239, 243 | 3 | 243-251 | - | - |
| Co75 | 2 | 180, 183 | 2 | 180， 183 | - | - |
| Co20 | 1 | 258 | 1 | 258 | 2 | 252， 258 |
| Co14 | 1 | 256 | 1 | 238 | 2 | 238， 244 |
| Co82 | 1 | 234 | 2 | 237, 240 | 2 | 237， 240 |
| Co43 | 1 | 250 | 2 | 246， 258 | - | - |
| Co22 | 3 | 257-265 | 2 | 261， 265 | 3 | 261-269 |
| Co228 | 2 | 239, 244 | 2 | 240， 244 | 1 | 244 |
| Co257 | 5 | 191-203 | 2 | 191， 200 | 2 | 191, 200 |
| Co66 | 1 | 205 | - | - | 1 | 205 |
| Co224 | 2 | 223, 229 | 2 | 223， 229 | 2 | 223， 229 |
| Co77 | 4 | 233-248 | - | - | 2 | 233， 236 |
| Co236 | 3 | 250-268 | 2 | 256， 259 | 2 | 256， 259 |
| Co222 | 3 | 198-223 | - | - | 2 | 217， 223 |
| Co244 | 2 | 269, 278 | - | - | 1 | 278 |
| Co234 | 2 | 191, 197 | 3 | 149-173 | - | - |
| Co261 | 2 | 267 | - | - | 2 | 261， 267 |
| Total | 27 |  | 20 |  | 22 |  |
